# Effect of respiratory motion correction and CT-based attenuation correction on dual-gated cardiac PET image quality and quantification

**DOI:** 10.1007/s12350-021-02769-6

**Published:** 2021-09-03

**Authors:** Jussi Schultz, Reetta Siekkinen, Mojtaba Jafari Tadi, Mika Teräs, Riku Klén, Eero Lehtonen, Antti Saraste, Jarmo Teuho

**Affiliations:** 1 Turku PET Centre, Turku University Hospital, Kiinamyllynkatu 4-8, 20521 Turku, Finland; 2 Turku PET Centre, University of Turku, Turku, Finland; 3 Department of Medical Physics, Turku University Hospital, Turku, Finland; 4 Department of Computing, University of Turku, Turku, Finland; 5 School of ICT, Turku University of Applied Sciences, Turku, Finland; 6 Heart Centre, Turku University Hospital, Turku, Finland; 7 Department of Biomedicine, University of Turku, Turku, Finland

## Abstract

**Background:**

Dual-gating reduces respiratory and cardiac motion effects but increases noise. With motion correction, motion is minimized and image quality preserved. We applied motion correction to create end-diastolic respiratory motion corrected images from dual-gated images.

**Methods:**

[^18^F]-fluorodeoxyglucose ([^18^F]-FDG) PET images of 13 subjects were reconstructed with 4 methods: non-gated, dual-gated, motion corrected, and motion corrected with 4D-CT (MoCo-4D). Image quality was evaluated using standardized uptake values, contrast ratio, signal-to-noise ratio, coefficient of variation, and contrast-to-noise ratio. Motion minimization was evaluated using myocardial wall thickness.

**Results:**

MoCo-4D showed improvement for contrast ratio (2.83 vs 2.76), signal-to-noise ratio (27.5 vs 20.3) and contrast-to-noise ratio (14.5 vs 11.1) compared to dual-gating. The uptake difference between MoCo-4D and non-gated images was non-significant (*P* > .05) for the myocardium (2.06 vs 2.15 g/mL), but significant (*P* < .05) for the blood pool (.80 vs .86 g/mL). Non-gated images had the lowest coefficient of variation (27.3%), with significant increase for all other methods (31.6-32.5%). MoCo-4D showed smallest myocardial wall thickness (16.6 mm) with significant decrease compared to non-gated images (20.9 mm).

**Conclusions:**

End-diastolic respiratory motion correction and 4D-CT resulted in improved motion minimization and image quality over standard dual-gating.

**Supplementary Information:**

The online version contains supplementary material available at 10.1007/s12350-021-02769-6.

## Introduction

Image quality in cardiac PET is reduced by respiratory and cardiac motions. Motion causes blurring and loss of details, reducing contrast and quantitative accuracy. To reduce motion effects, cardiac- or respiratory gating can be applied. An image consisting of the diastolic phase can be reconstructed to minimize cardiac motion.^1^ To minimize respiratory and cardiac motions simultaneously, dual-gating was proposed,^2^,^3^ which minimizes motion but increases noise as less data are used for reconstruction.

To improve image quality in dual-gated PET, motion correction has been proposed.^4^ Motion correction can be applied as respiratory-only, cardiac-only or a combination of the two. These approaches correct and combine respiratory or cardiac phases to a reference phase, usually the end-diastole or end-expiratory phase. Correction is performed via deformation and registration using reconstructed images or during reconstruction. Motion correction increases signal-to-noise ratio, myocardium-to-blood contrast and reduces myocardial thickness, resulting in high quality and motion-frozen PET images.^5^

However, motion correction has not gained widespread clinical utility, due to complexity of implementation. Lamare et al. compared multiple schemes for motion correction,^6^ proposing to use the diastolic phase with respiratory motion correction as a simple, clinically viable methodology. Thus, the approach in^6^ could offer improved image quality and similar motion compensation as dual-gating.

Moreover, motion effects on CT-based attenuation correction (CTAC) should be minimized, such as misalignment^7^-^9^ and the difference in temporal resolution between PET and CT.^10^,^11^ To circumvent these, two methods have been proposed. The first is creating an average CT image from cinematic CT over respiratory cycles to match the temporal resolutions.^9^ The second is to “gate” the CT acquisition, matching the respiratory cycles between CT and PET.^12^

In this study, the clinical applicability of the approach in Ref. 6 with fully quantitative PET data are shown. By creating an end-diastolic, respiratory motion corrected PET image, effective motion minimization and improved image quality compared to dual-gating are achieved. Moreover, we use motion correction available on clinical PET/CT system and evaluate the method with respiratory-gated CT (4D-CT) and cinematic CT (CINE-CT), assessing their effect to the accuracy of motion correction, for the first time.

## Materials and Methods

### PET/CT System

Data were acquired on GE Discovery 690 (D690) PET/CT (GE Healthcare, US). D690 is a 3D-PET system containing a 64-slice Lightspeed CT. The performance evaluation with technical details is reported in Ref. 13. Transaxial and axial fields of view (FOV) are 70 cm and 15.7 cm. The coincidence timing and energy windows are 4.9 ns and 425-650 keV.

### Data Acquisition

We recruited 13 subjects undergone [^18^F]-fluorodeoxyglucose ([^18^F]-FDG) PET/CT. Population characteristics are in Table 1. Imaging was performed after an acute coronary syndrome or percutaneous coronary intervention due to chronic coronary artery disease, to evaluate inflammation in the coronary arteries. A high-fat non-carbohydrate diet was started 24 hours before imaging with extended fasting of 8 hours. Patients were on beta-blocker medication. Intravenous metoprolol 0 to 30 mg was administered before coronary angiography to reach a heart rate of < 60 bpm. The study was approved by the ethics committee of the Hospital District of Southwest Finland and Turku University Hospital (ETMK 44/180/2012). Each subject gave an informed consent.

**Table 1:** Subject characteristics with medians and interquartile ranges, unless otherwise indicated

Demographics	Value
Age (years)	59.0 (53.5–65.5)
Male, N (%)	11 (85)
Weight (kg)	91.0 (76.5–100.5)
Height (m)	1.75 (1.70–1.80)
BMI (kg/m^2^)	28.0 (25.6–30.8)
Dose (MBq)	305 (298–311)

PET was acquired in list-mode for one bed position over the heart, after an uptake period of 90 minutes. 3D-PET with simultaneous respiratory- and cardiac gating with 24 minutes duration was acquired. Respiratory position monitor (RPM) system (Varian Medical Systems, US) was used for respiratory tracking. A standard 3-lead ECG system (IVY-3150, Ivy Biomedical, US) integrated with the PET/CT hardware was used for cardiac gating.

After PET, a 4D respiratory-gated CINE-CT was acquired over the same bed position. The parameters were: cinematic mode; slice thickness 2.5 mm; cine time .45 seconds; rotation time .5 seconds; tube voltage 120 kV; current 30 mA s. The average cine duration was 4.71 ± 1.63 seconds. The radiation dose for CINE-CT and PET were 2.67 mSv and 5.90 mSv on average. During CT and PET, the subjects were instructed to breathe normally.

### PET Reconstruction and Dual-Gating

**Figure 1:**
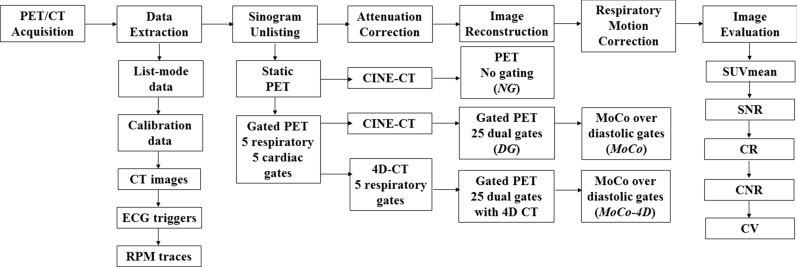
Image reconstruction and registration pipeline. 25 dual-gated PET images were reconstructed, with CINE-CT and 4D-CT. Motion correction was performed to respiratory bins containing the diastolic phase, using bin numbers 21 to 25

Image reconstruction and registration pipeline are shown in Figure 1. List-mode data, CT images, and respiratory curves during PET and CT were exported from the RPM system. All data were processed using vendor-provided reconstruction and gating toolbox (RGT) and in-house software, implemented using MATLAB2015b. Three sets of PET images were reconstructed: (1) non-gated images with CINE-CT, (2) dual-gated with CINE-CT (3) dual-gated with 4D-CT. Respiratory motion correction was applied to dual-gated images over the respiratory bins containing the diastolic phase of the heart.

Non-gated images were reconstructed in static mode using full 24 minutes of acquisition time. For dual-gating, 5 respiratory and 5 cardiac bins were used, producing a total of 25 individual dual-gated bins. A long end-diastolic, end-expiratory gate for optimal count statistics and motion minimization was preserved in bin number 25, which was used as a reference phase for motion correction and evaluation of dual-gated images. Respiratory motion correction was applied to bin numbers 21 to 25.

For respiratory gating in PET and CT, amplitude gating was applied. Amplitude gating is effective with irregular breathing^14^ and saves ~30% data in the end-expiratory bin. The bins were divided equally by amplitude from end-inspiration to end-expiration. The gating thresholds were determined by equidistant sampling. The maximum threshold was defined as mean plus one standard deviation of the amplitude maxima, whereas the minimum was determined from the mean of the amplitude minima. Only the cycles considered as “valid cycles” by the RPM were used.

For cardiac gating (ECG-gating), 5 bins using the cardiac triggers from list-mode data were defined. ECG-gating was optimized to produce ~ 35% data in end-diastolic bin. Non-equidistant, fixed-time gate assignments between subsequent R-peaks were used. The time division of cardiac cycles was: 50, 120, 420, 550 and 1500 ms from the R-peak. Fixed-time gating was used due to premedication and relatively stable average heart rate, which was 61.95 ± 11.71 bpm during PET. This ensured similar gating intervals and statistics with a large diastolic gate throughout all subjects.

Attenuation correction was performed with CINE-CT and 4D-CT. For CINE-CT, the CT images were averaged over all respiratory phases and used for attenuation correction of dual-gated and non-gated PET. Another set of dual-gated images were reconstructed using amplitude gated 4D-CT, using the RPM respiratory curve and exported CT images. CT-PET alignment was inspected over different respiratory phases.

PET images were reconstructed with three-dimensional ordered subsets expectation maximization (3D-OSEM) algorithm, using 2 iterations, 24 subsets and a 6 mm Gaussian post-filter. The matrix and FOV size were 256 × 256 × 47 and 350 mm. Resolution recovery (PSF correction) was applied as recommended in Ref. 5. All quantitative corrections including decay, attenuation, scatter and randoms were applied.

### Motion Correction

Motion correction was implemented based on Ref. 6. All respiratory gates of the dual-gated images over the diastolic phase were registered post-reconstruction using the end-expiratory, end-diastolic phase as the reference phase. A respiratory motion corrected end-diastolic PET image was then created using gates from bins 21 (peak-inspiration, diastolic) to 25 (end-expiration, diastolic).

A level-sets based non-rigid registration algorithm^15^ was used, with the implementation described in Ref. 16. Three levels with 10, 10 and 30 iterations per individual level were applied. The registration update fields were regularized after each iteration using a 3D-Gaussian kernel with *σ* = .2. The moving images were smoothed with a Gaussian kernel of 1 mm. The registration accuracy was confirmed visually.

The following PET images were created: non-gated with CINE-CT (**NG**), dual-gated corresponding to the end-expiratory, end-diastolic phase (bin number 25) with CINE-CT (**DG**), motion corrected from dual-gated images with CINE-CT (**MoCo**) and motion corrected from dual-gated images with 4D-CT (**MoCo-4D**), both corresponding to respiratory motion corrected images from end-diastolic phase, combined from bins 21 to 25.

## Image Processing and Analysis

### Data Preservation

The amount of data preserved compared to the NG image (100%) was calculated. For DG, this was evaluated from the end-diastolic, end-expiratory bin. For MoCo and MoCo-4D, motion corrected images were used.

### Computational Speed

Computational effectiveness was evaluated on a 2.27 GHz Intel Xeon (L5640), 16 GB of memory, 64-bit Windows 7 and MATLAB2015b. We evaluated the speed of whole motion correction pipeline and the motion correction process only, by excluding file reading and writing. The average of 5 runs is reported.

### Image Analysis

Images were analyzed using Carimas 2.9 (Turku PET Centre, Finland), with automatic volume of interest (VOI) placement in the left ventricle and inside the left ventricle blood pool. For 3 subjects, the VOIs over the myocardia were manually smoothed to improve segmentation quality.

Myocardial VOI volumes ranged between 94.0 and 226 cm^3^ with a mean volume of 150 ± 34.1 cm^3^. The blood pool VOI volumes were 6.82-16.6 cm^3^ with a mean volume of 11.0 ± 2.28 cm^3^. Mean standardized uptake values (SUV_mean_) and standard deviations of the SUV (SUV_SD_) in the myocardium and blood pool VOI were determined.

From the SUV, following image quality metrics were calculated: contrast ratio (CR), signal-to-noise ratio (SNR), coefficient of variation (CV), and contrast-to-noise ratio (CNR), which reflect image quality and have been previously used in evaluation of motion correction.^5^,^6^,^17^

The metrics were calculated as:

1 $$ {\text{CR}} = \frac{{{\text{SUV}}_{{{\text{mean}}}} \;{\text{in}}\;{\text{the}}\;{\text{myocardium}}}}{{{\text{SUV}}_{{{\text{mean}}}} \;{\text{in}}\;{\text{the}}\;{\text{blood}}\;{\text{pool}}}}, $$

2 $$ {\text{SNR}} = \frac{{{\text{SUV}}_{{{\text{mean}}}} \;{\text{in}}\;{\text{the}}\; {\text{myocardium}}}}{{{\text{SUV}}_{{{\text{SD}}}} \;{\text{in}}\;{\text{the}}\;{\text{blood}}\;{\text{pool}}}}, $$

3 $$ {\text{CV}} = \frac{{{\text{SUV}}_{{{\text{SD}}}} \;{\text{in}}\;{\text{the}}\;{\text{myocardium}}}}{{{\text{SUV}}_{{{\text{mean}}}} \;{\text{in}}\;{\text{the}}\;{\text{myocardium}}}}, $$

4 $$ {\text{CNR}} = \frac{{{\text{SUV}}_{{{\text{mean}}}} \;{\text{in}}\;{\text{the}}\;{\text{myocardium}} - {\text{SUV}}_{{{\text{mean}}}} \;{\text{in}}\;{\text{the}}\;{\text{blood}}\,{\text{pool}}}}{{{\text{SUV}}_{{{\text{SD}}}} \;{\text{in}}\;{\text{the}}\;{\text{blood}}\;{\text{pool}}}}. $$

Motion minimization performance was evaluated using myocardial wall thickness (MWT) of the left ventricle. MWT was determined as the full width at half maximum of the uptake profile across the antero-lateral myocardial wall, measured in the short-axis view at the midventricular level.

### Statistical Analysis

Statistical analyses were performed using JMP Pro Version 13.1.0 (SAS Institute Inc., US). Continuous variables are reported as mean±standard deviation if normally distributed, and as median (interquartile range) otherwise. Normality was tested by visual inspection and using the Shapiro-Wilk test. Categorical variables are reported as N (%). Differences in image quality metrics were compared using Wilcoxon’s signed rank test. All statistical tests were two-tailed, with *P*-values < .05 denoting statistical significance.

## Results

### Data Preservation

The amount of data preserved was 38.98 ± 5.28% in the MoCo and MoCo-4D images, and 10.38 ± 3.20% in the DG images. Excluding non-valid cycles resulted in average data loss of 13.84 ± 5.76%, with the largest and smallest loss of 28.15% and 5.77%.

### Computational Speed

The correction process lasted 290 seconds and was increased to 333 seconds when running the whole pipeline.

### Image Quality and Quantification

#### Standardized uptake values

**Figure 2:**
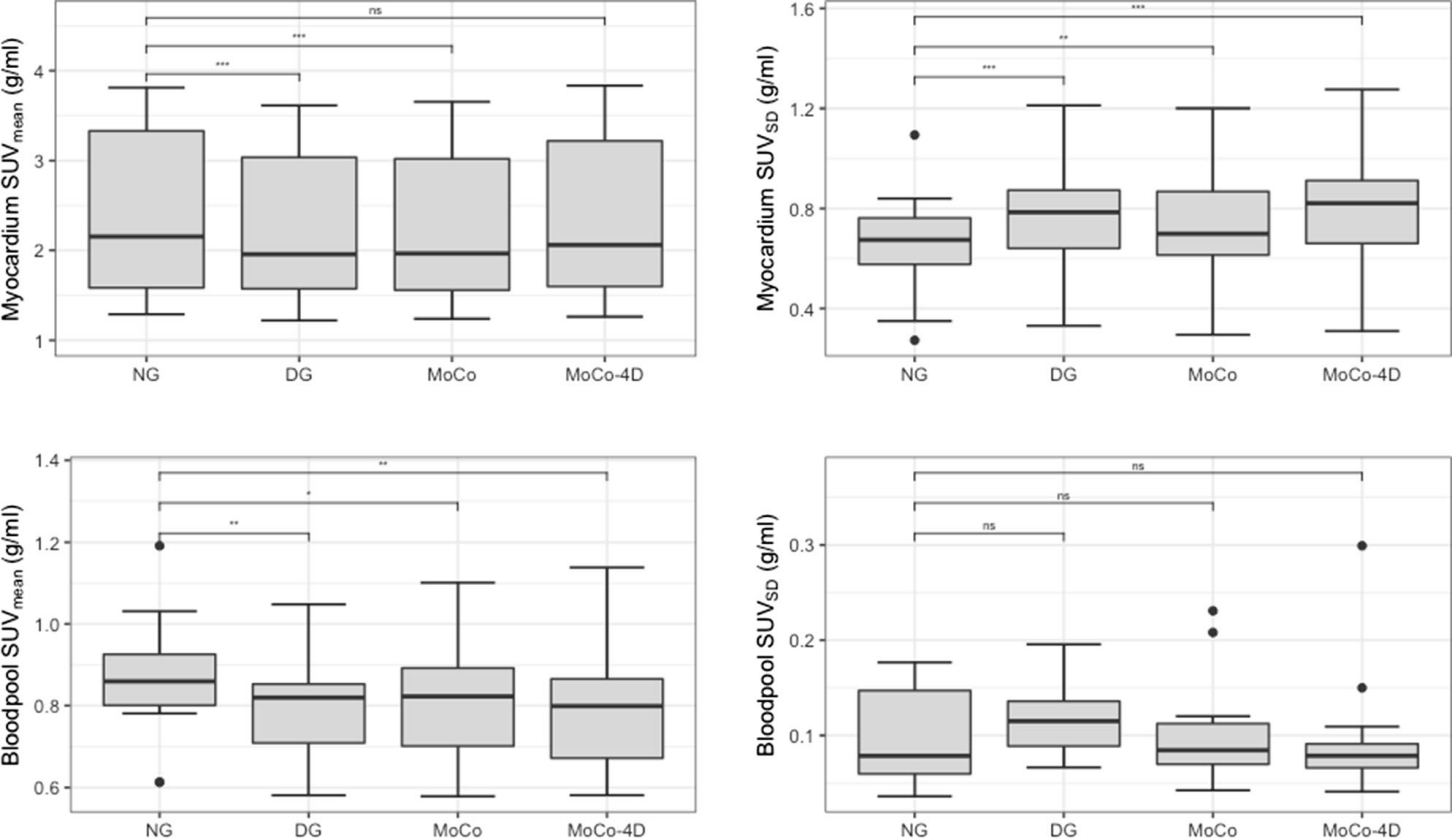
Standardized uptake values (SUV_mean_, SUV_SD_) of the myocardium and blood pool (ns = non-significant, *P < .05, **P < .01 ***P < .001)

Table 2 contains the SUV_mean_ with statistical comparisons versus DG. SUV results are summarized in Figure 2 with statistical comparisons versus NG.

**Table 2:** SUV_mean_ for the myocardium and blood pool

	Myocardium SUV_mean_ (g/mL)	Blood pool SUV_mean_ (g/mL)
NG	2.15 (1.48–3.43)	.86 (.79–.95)
DG	1.96 (1.46–3.17)	.82 (.71–.87)
MoCo	1.96 (1.46–3.16)	.82 (.69–.90)
MoCo-4D	2.06* (1.52–3.30)	.80 (.67–0.87)

Medians and interquartile ranges are reported

*Denotes statistically significant difference (*P* < .05) between DG and MoCo-4D

NG had the highest myocardium SUV_mean_ of 2.15 g/mL. DG and MoCo had comparable SUV_mean_ of 1.96 g/mL. MoCo-4D SUV_mean_ was 2.06 g/mL, with significant improvement compared to DG and MoCo images. The difference between NG and MoCo-4D images was non-significant.

NG had the highest blood pool SUV_mean_ of .86 g/mL. DG and MoCo images had slightly lower values of .82 g/mL. MoCo-4D had the lowest SUV_mean_ with .80 g/mL. All pairwise differences between DG, MoCo and MoCo-4D were non-significant. All methods showed significant decrease compared to NG images.

**Figure 3:**
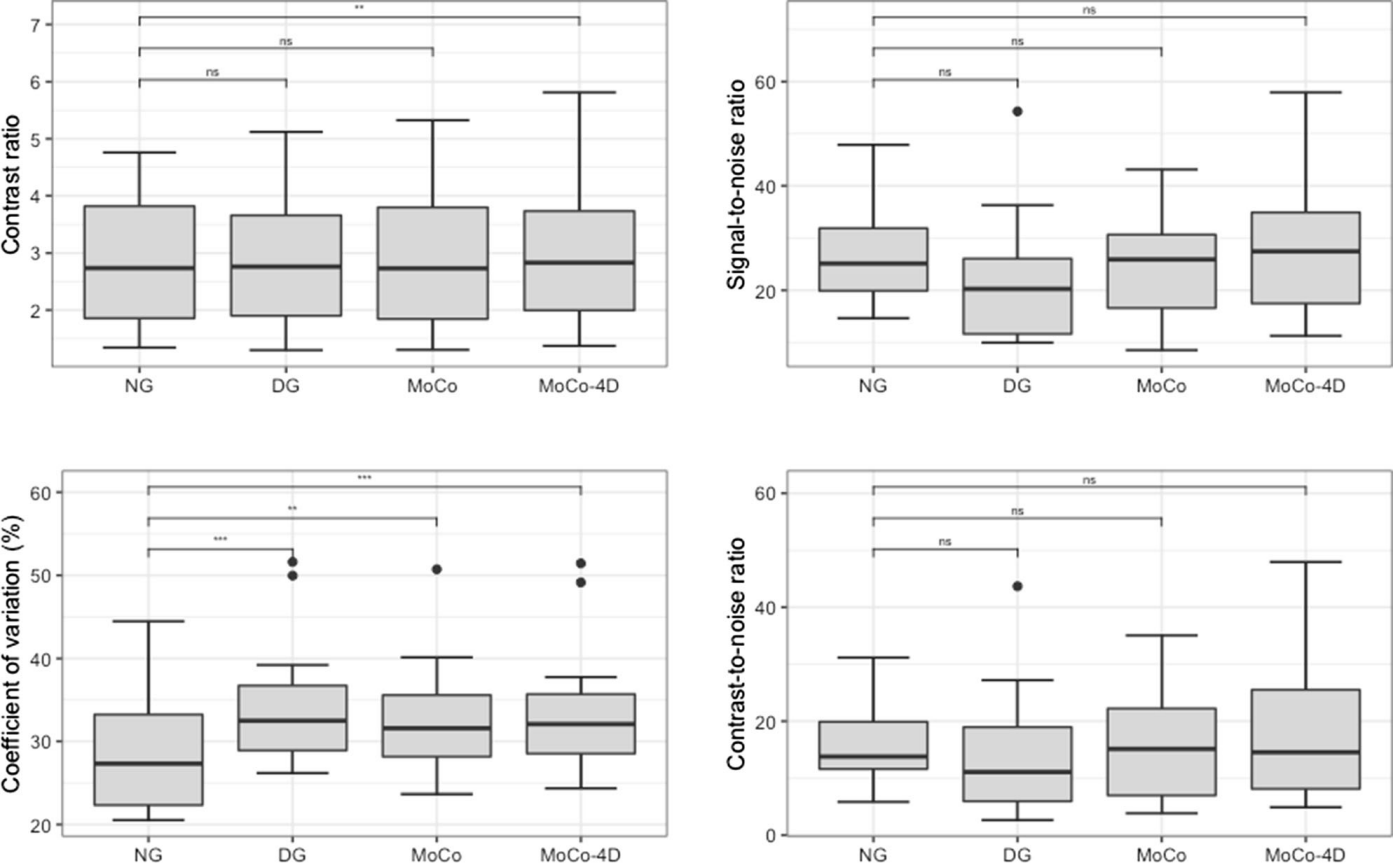
Image quality metrics (ns=non-significant, *P < .05, **P < .01 ***P < .001)

**Figure 4:**
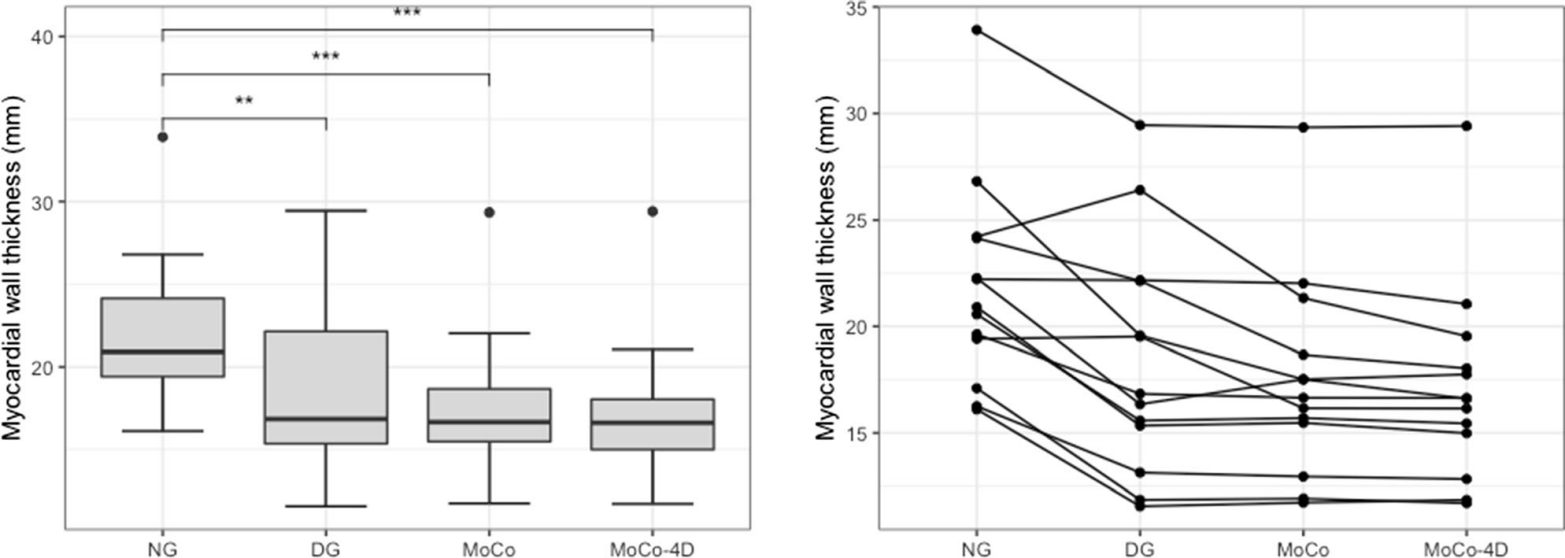
On the left, myocardial wall thickness (MWT) is shown (**P < .01, ***P < .001). On the right, MWT is shown for each subject. MoCo and MoCo-4D show lowest MWT

Table 3 contains all image quality metrics. Figure 3 contains box and whisker plots of CR, SNR, CV and CNR with statistical comparisons. MWT for all methods and subjects is illustrated in Figure 4.

**Table 3:** Image quality metrics

	CR	SNR	CV (%)	CNR	MWT (mm)
NG	2.74 (1.85–3.83)	25.1 (19.4–34.4)	27.3 (22.2–34.3)	13.8 (10.0–22.5)	20.9 (18.3–24.2)
DG	2.76 (1.87–3.83)	20.3 (11.4–27.7)	32.5 (28.8–38.0)	11.1 (5.57–20.7)	16.8 (14.2–22.2)
MoCo	2.73 (1.82–3.88)	25.9 (16.2–31.2)	31.6 (28.0–36.7)	15.1 (6.58–22.9)	16.7 (14.2–20.0)
MoCo-4D	2.83* (1.93–4.03)	27.5* (16.9–39.3)	32.1* (28.4–36.7)	14.5* (7.77–30.1)	16.6* (13.9–18.8)

Medians and interquantile ranges are reported

*Denotes statistically significant difference (*P* < .05) between DG and MoCo-4D

### Contrast Ratio

NG images had a CR of 2.74, whereas DG and MoCo images had CR of 2.76 and 2.73. MoCo-4D had superior CR of 2.83 with statistically significant increase compared to other methods.

### Signal-to-Noise Ratio

NG images had SNR of 25.1. The lowest SNR was seen in DG images (20.3), with MoCo in between (25.9). MoCo-4D images had highest SNR of 27.5. Compared to NG images, the difference was non-significant. Significant improvement was observed in MoCo-4D images compared to DG and MoCo images.

### Coefficient of Variation

NG images had lowest CV of 27.3%. CV increased for all other methods, with values of 32.5% for DG, 31.6% for MoCo, and 32.1% for MoCo-4D. All differences were statistically significant.

### Contrast-to-Noise Ratio

NG images had a CNR of 13.8, whereas DG images had CNR of 11.1. MoCo and MoCo-4D images had the highest CNR of 15.1 and 14.5. The difference between MoCo-4D and DG was significant. The median change from MoCo to MoCo-4D was significant with increase of 1.85 (*P* = .0327, 95% CI − .073-11.02).

### Myocardial Wall Thickness

Highest MWT was measured in NG images (20.9 mm). All other methods showed significant decrease compared to NG. DG and MoCo had MWT of 16.8 mm and 16.7 mm, with MoCo-4D having the lowest MWT of 16.6 mm and lowest interquartile range. The median decrease in MoCo-4D compared to DG and MoCo was significant.

### Visual Quality

**Figure 5:**
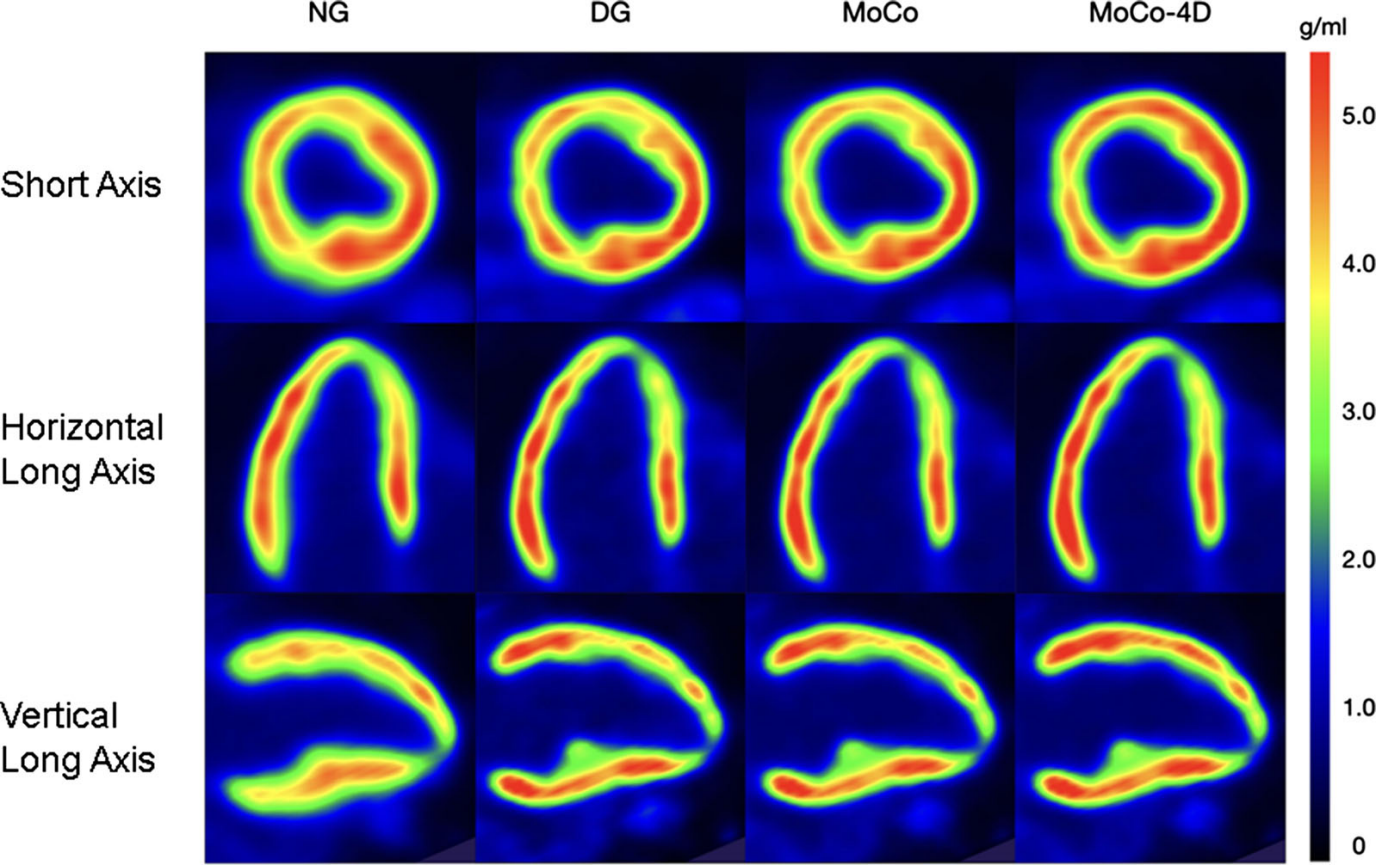
PET images for patient A (female, 54 years, 170 cm, 81 kg, BMI 28.0, 305 MBq)

**Figure 6:**
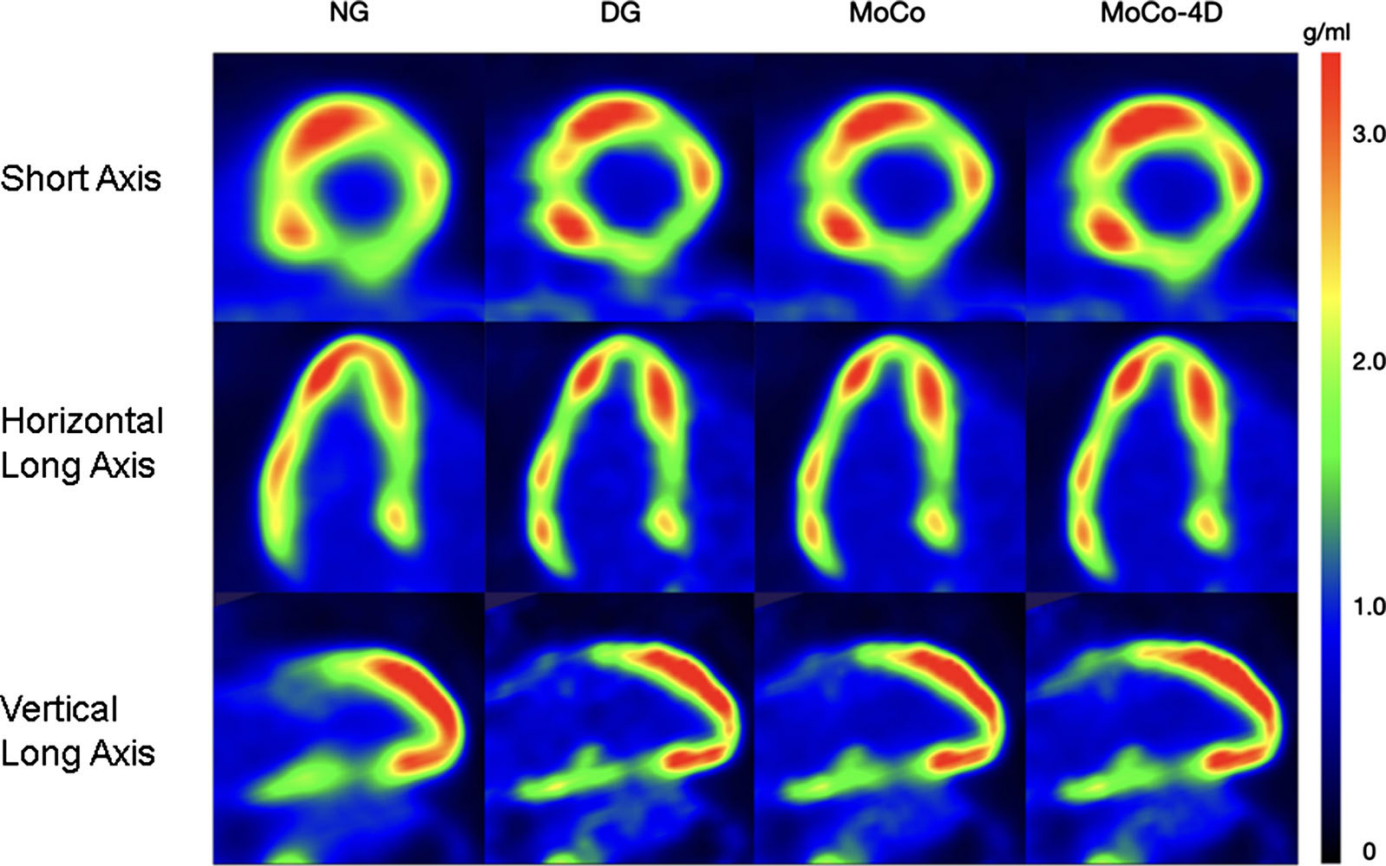
PET images for patient B (male, 54 years, 171 cm, 73 kg, BMI 25.0, 298 MBq)

Figures 5 and 6 visualize the myocardium and blood pool in two subjects, demonstrating image quality between the methods and reduced spill-over compared to NG.

**Figure 7:**
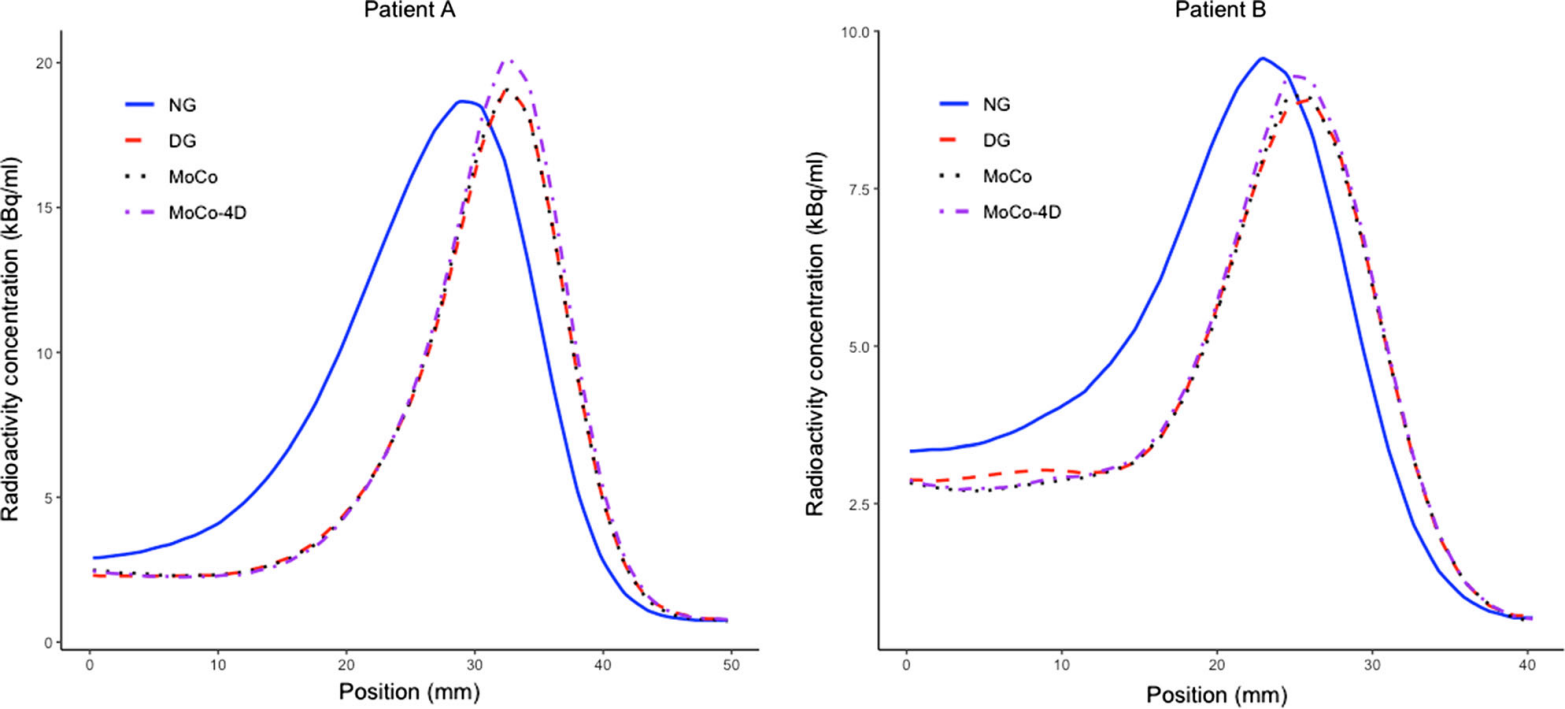
Uptake profiles across the antero-lateral myocardial wall for patients A (Fig. 5) and B (Fig. 6), showing reduced MWT. The peak activity is slightly lower for motion correction with patient B

Figure 7 shows the uptake profiles used to calculate MWT with the same subjects.

## Discussion

The creation of a respiratory motion corrected end-diastolic image (MoCo-4D) minimizes motion and improves image quality compared to dual-gating. This method also saves 25% of additional data compared to dual-gating. The proposed methods could be implemented using existing software on a clinical PET/CT system.

### Method Evaluation

MoCo-4D was superior in majority of the image quality metrics with optimum motion minimization, showing statistically significant improvement compared to both NG and DG.

MoCo-4D showed increased myocardial SUV_mean_ and lower blood pool SUV_mean_ compared to other methods (Table 2, Figure 2). MoCo-4D showed improved SUV_mean_ recovery compared to DG and MoCo and reduced spill-over compared to NG.

In terms of image quality (Table 3, Figure 3), MoCo-4D showed superiority in a majority of measured parameters compared to DG. For CR and SNR, this can be seen clearly.

CV increased in DG, MoCo and MoCo-4D to similar level (~32-33%). For DG, this is caused by increase of noise. As motion correction is performed post-reconstruction, noise is averaged in the corrected image, increasing CV in MoCo and MoCo-4D.

MoCo-4D reduced motion compared to NG, with 16.6 mm vs 20.9 mm MWT (Table 3). MoCo and DG images had similar MWT (16.6-16.8 mm), indicating that no residual blurring was introduced by MoCo. MoCo-4D resulted in slightly smaller MWT subject-wise (Figure 4).

In the visual evaluation, spill-over in the blood pool was reduced with DG and MoCo methods (Figures 5 and 6). Slight increases in local uptake were visible in MoCo methods, with no additional blurring compared to DG. The uptake profiles were also sharper due to motion minimization (Figure 7).

### Comparison of 4D-CT and CINE-CT

We compared two methods for attenuation correction. Using CINE-CT in cardiac imaging improves accuracy.^8^,^9^ We have shown that motion correction benefits from 4D-CT with the same radiation dose as CINE-CT. However, CINE-CT increases dose and might not be justified, if no benefits are seen over the standard CTAC protocol.^18^

### Comparison to Previous Studies

MoCo-4D has higher MWT (16.6 mm) than 13.8 mm reported in Ref. 5 and 11.6 mm in Ref. 17, which applied cardiac^5^ and cardiac-respiratory^17^ motion correction. We used a 6 mm Gaussian filter from our clinical protocol, which is optimal for noise suppression but reduces resolution. A 2 mm filter used in Refs. 11,17 might be more optimal. As in previous studies, we noted increased CR and CNR, although direct comparison is challenging due to different methodologies.

A trade-off exists between motion minimization and noise increase in dual-gating, degrading registration accuracy.^6^ We applied 5/5 cardiac and respiratory bins according to our clinical protocol versus 8/4 in Ref. 6. This is nearly identical to the optimal amount reported in Ref. 19, although on a different system. A more optimal amount might be discovered with systematic testing, which is out of the scope of this study.

### Limitations

Differences in SUV_mean_ and CV are attributed to VOI delineation, which was performed independently for each image. This causes variability in VOI size and the activity covered by the VOI, increasing standard deviation and CV. We quantified SUV_mean_ instead of SUV_max_, since the dependence of SUV_max_ on counting statistics has been shown^20^ whereas SUV_mean_ is more robust.^21^ The method was also evaluated with a small number (N = 13) of patients, prompting follow-up studies with larger patient groups.

Motion correction is challenging in plaque imaging. All subjects in this study had myocardial uptake, thus the motion correction performance with coronary lesions of low uptake was not evaluated. A level set-based registration has been applied in [^18^F]-NaF PET,^22^ using coronary CT angiography for PET registration.

### Future Studies

Our method saves 39% of the data, as only the diastolic phase is used for motion correction. Thus, a dual motion correction approach as in Ref. 17 might be preferred, which can preserve 100% of the data. Applying cardiac motion correction as in Ref. 23 might also improve image quality and MWT, however, these approaches are not yet implemented on PET/CT systems. Moreover, a triple-motion correction including gross patient motion correction might increase performance.^21^

Finally, sinogram-based motion correction is advantageous for image quality and motion minimization. A challenge has been adopting sinogram-based methods in clinical routine, due to complexity and user-defined reconstruction, challenging clinical comparison.^24^ However, one such approach has been recently implemented using vendor-based reconstruction.^24^ Thus, modifying our method to sinogram-based correction could improve performance.

### New Knowledge Gained

We implemented a motion correction method which corrects respiratory motion over the diastolic phase combined with 4D-CT. The method improves image quality and motion minimization qualities significantly and is relatively simple to implement clinically using existing methodologies. We’ve shown the clinical applicability of the proposed approach and that 4D-CT should be used in combination of motion correction for optimum results.

## Conclusions

This paper proposed a motion correction approach that could be implemented using existing vendor-based methods in cardiac PET/CT imaging. The best motion minimization qualities together with the best image quality and quantification metrics were achieved with the MoCo-4D method.

## Supplementary Information

Below is the link to the electronic supplementary material.

Supplementary file1 (PPTX 256 kb)

## Abbreviations

4D-CT: Respiratory-gated computed tomography
CINE-CT: Cinematic computed tomography
CNR: Contrast to noise ratio
CR: Contrast ratio
CV: Coefficient of variation
DG: Dual-gated
MoCo: Motion correction
MoCo-4D: Motion correction with respiratory-gated computed tomography
MWT: Myocardial wall thickness
NG: Non-gated

## References

[1] Joshi NV, Vesey AT, Williams MC, Shah ASV, Calvert PA, Craighead FHM (2014). 18F-fluoride positron emission tomography for identification of ruptured and high-risk coronary atherosclerotic plaques: a prospective clinical trial. Lancet.

[2] Kokki T, Sipilä HT, Teräs M, Noponen T, Durand-Schaefer N, Klén R (2010). Dual gated PET/CT imaging of small targets of the heart: method description and testing with a dynamic heart phantom. J Nucl Cardiol.

[3] Teräs M, Kokki T, Durand-Schaefer N, Noponen T, Pietilä M, Kiss J (2010). Dual-gated cardiac PET-clinical feasibility study. Eur J Nucl Med Mol Imaging.

[4] Rubeaux M, Doris MK, Alessio A, Slomka PJ (2017). Enhancing cardiac PET by motion correction techniques. Curr Cardiol Rep.

[5] Le Meunier L, Slomka PJ, Dey D, Ramesh A, Thomson LEJ, Hayes SW (2011). Motion frozen 18F-FDG cardiac PET. J Nucl Cardiol.

[6] Lamare F, Le Maitre A, Dawood M, Schäfers KP, Fernandez P, Rimoldi OE (2014). Evaluation of respiratory and cardiac motion correction schemes in dual gated PET/CT cardiac imaging. Med Phys.

[7] Chin BB, Nakamoto Y, Kraitchman DL, Marshall L, Wahl R (2003). PET-CT evaluation of 2-deoxy-2-[18F]fluoro-D-glucose myocar-dial uptake: Effect of respiratory motion. Mol Imaging Biol.

[8] Gould KL, Pan T, Loghin C, Johnson NP, Guha A, Sdringola S (2007). Frequent diagnostic errors in cardiac PET/CT due to misregistra-tion of CT attenuation and emission PET images: A definitive analysis of causes, consequences, and corrections. J Nucl Med.

[9] Pan T, Mawlawi O, Luo D, Liu HH, Chi P-CM, Mar MV (2006). Attenuation correction of PET cardiac data with low-dose average CT in PET/CT. Med Phys.

[10] Le Meunier L, Maass-Moreno R, Carrasquillo JA, Dieckmann W, Bacharach SL (2006). PET/CT imaging: Effect of respiratory motion on apparent myocardial uptake. J Nucl Cardiol.

[11] Pan T (2018). Respiratory gating in PET/CT: A step in the right direction. J Nucl Cardiol.

[12] Pan T, Lee T-Y, Rietzel E, Chen GTY (2004). 4D-CT imaging of a volume influenced by respiratory motion on multi-slice CT. Med Phys.

[13] Bettinardi V, Presotto L, Rapisarda E, Picchio M, Gianolli L, Gilardi MC (2011). Physical performance of the new hybrid PET/CT Discovery-690. Med Phys.

[14] Dawood M, Büther F, Lang N, Schober O, Schäfers KP (2007). Respiratory gating in positron emission tomography: A quantitative comparison of different gating schemes. Med Phys.

[15] Vemuri BC, Ye J, Chen Y, Leonard CM (2003). Image registration via level-set motion: Applications to atlas-based segmentation. Med Image Anal.

[16] Wollenweber SD, Gopalakrishnan G, Thielemans K, Manjeshwar RM (2012). Evaluation of the accuracy and robustness of a motion correction algorithm for PET using a novel phantom approach. IEEE Trans Nucl Sci.

[17] Slomka PJ, Rubeaux M, Le Meunier L, Dey D, Lazewatsky JL, Pan T (2015). Dual-gated motion-frozen cardiac PET with Flurpiridaz F 18. J Nucl Med.

[18] Tzolos E, Lassen ML, Pan T, Kwiecinski J, Cadet S, Dey D, et al. Respiration-averaged CT versus standard CT attenuation map for correction of 18F-sodium fluoride uptake in coronary atherosclerotic lesions on hybrid PET/CT. J Nucl Cardiol 2020 (in press). 10.1007/s12350-020-02245-7 PMC7775905 32617857

[19] Klén R, Teuho J, Noponen T, Thielemans K, Hoppela E, Lehtonen E (2020). Estimation of optimal number of gates in dual gated 18 F-FDG cardiac PET. Sci Rep.

[20] Liu C, Alessio A, Pierce L, Thielemans K, Wollenweber S, Ganin A (2010). Quiescent period respiratory gating for PET/CT. Med Phys.

[21] Lassen ML, Kwiecinski J, Dey D, Cadet S, Germano G, Berman DS (2019). Triple-gated motion and blood pool clearance corrections improve reproducibility of coronary 18F-NaF PET. Eur J Nucl Med Mol Imaging.

[22] Rubeaux M, Joshi NV, Dweck MR, Fletcher A, Motwani M, Thomson LE (2016). Motion correction of 18F-NaF PET for imaging coronary atherosclerotic plaques. J Nucl Med.

[23] Slomka PJ, Nishina H, Berman DS (2004). “Motion-frozen” display and quantification of myocardial perfusion. J Nucl Med.

[24] Lassen ML, Beyer T, Berger A, Beitzke D, Rasul S, Büther F (2020). Data-driven, projection-based respiratory motion compensation of PET data for cardiac PET/CT and PET/MR imaging. J Nucl Cardiol.

