# Supplementary material for: Effect of respiratory motion correction and CT-based attenuation correction on dual-gated cardiac PET image quality and quantification
Source: J Nucl Cardiol. 2021 Sep 3;29(5):2423–33. doi: 10.1007/s12350-021-02769-6 (PMC9553777; doi:10.1007/s12350-021-02769-6)
Supplement: Supplementary file 1 — Supplementary file1 (PPTX 256 kb) [file 12350_2021_2769_MOESM1_ESM.pptx]

## Slide 1
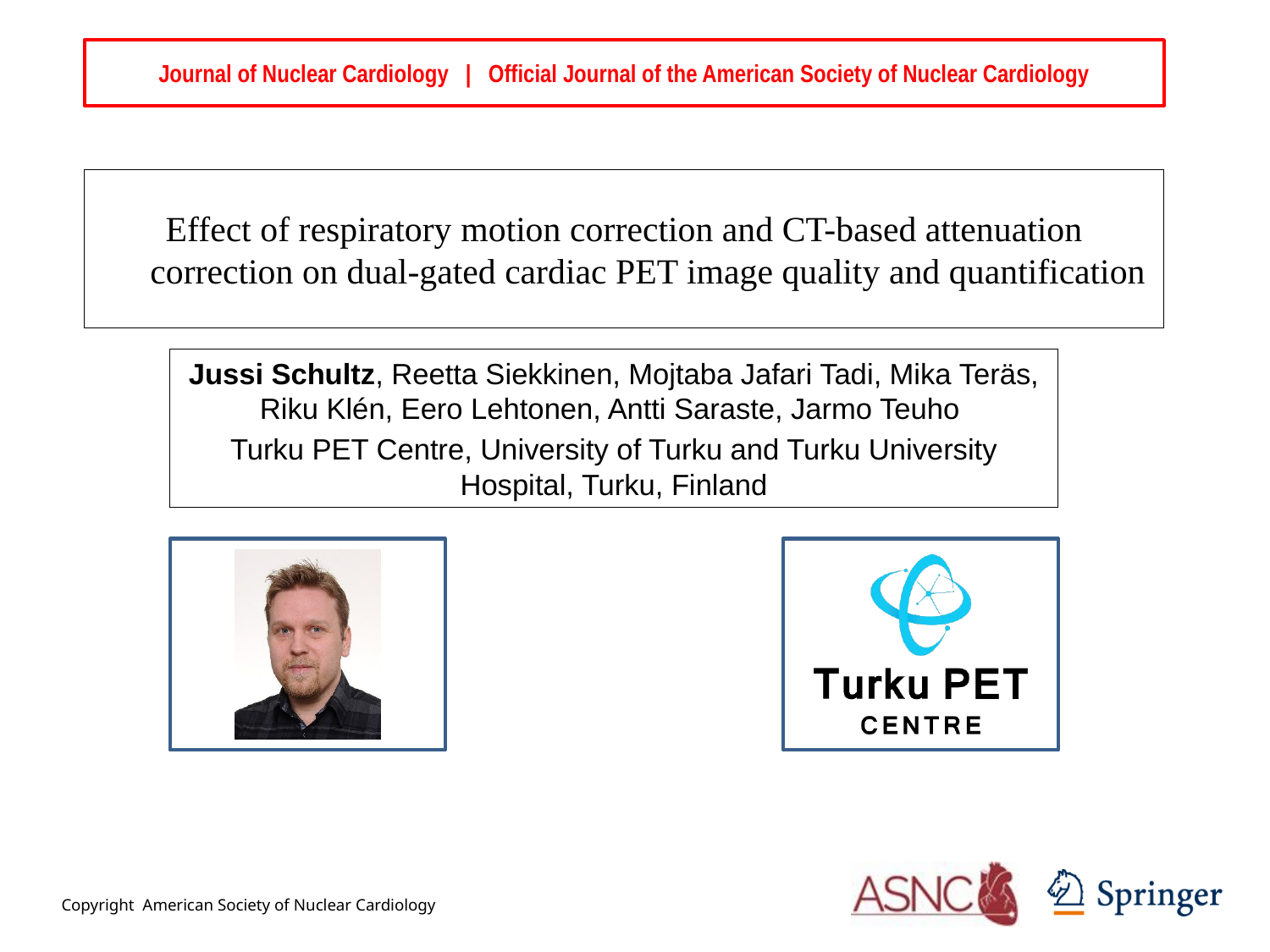

Journal of Nuclear Cardiology | Official Journal of the American Society of Nuclear Cardiology
# Effect of respiratory motion correction and CT-based attenuation correction on dual-gated cardiac PET image quality and quantification
Jussi Schultz, Reetta Siekkinen, Mojtaba Jafari Tadi, Mika Teräs, Riku Klén, Eero Lehtonen, Antti Saraste, Jarmo Teuho
Turku PET Centre, University of Turku and Turku University Hospital, Turku, Finland
Copyright American Society of Nuclear Cardiology

## Slide 2
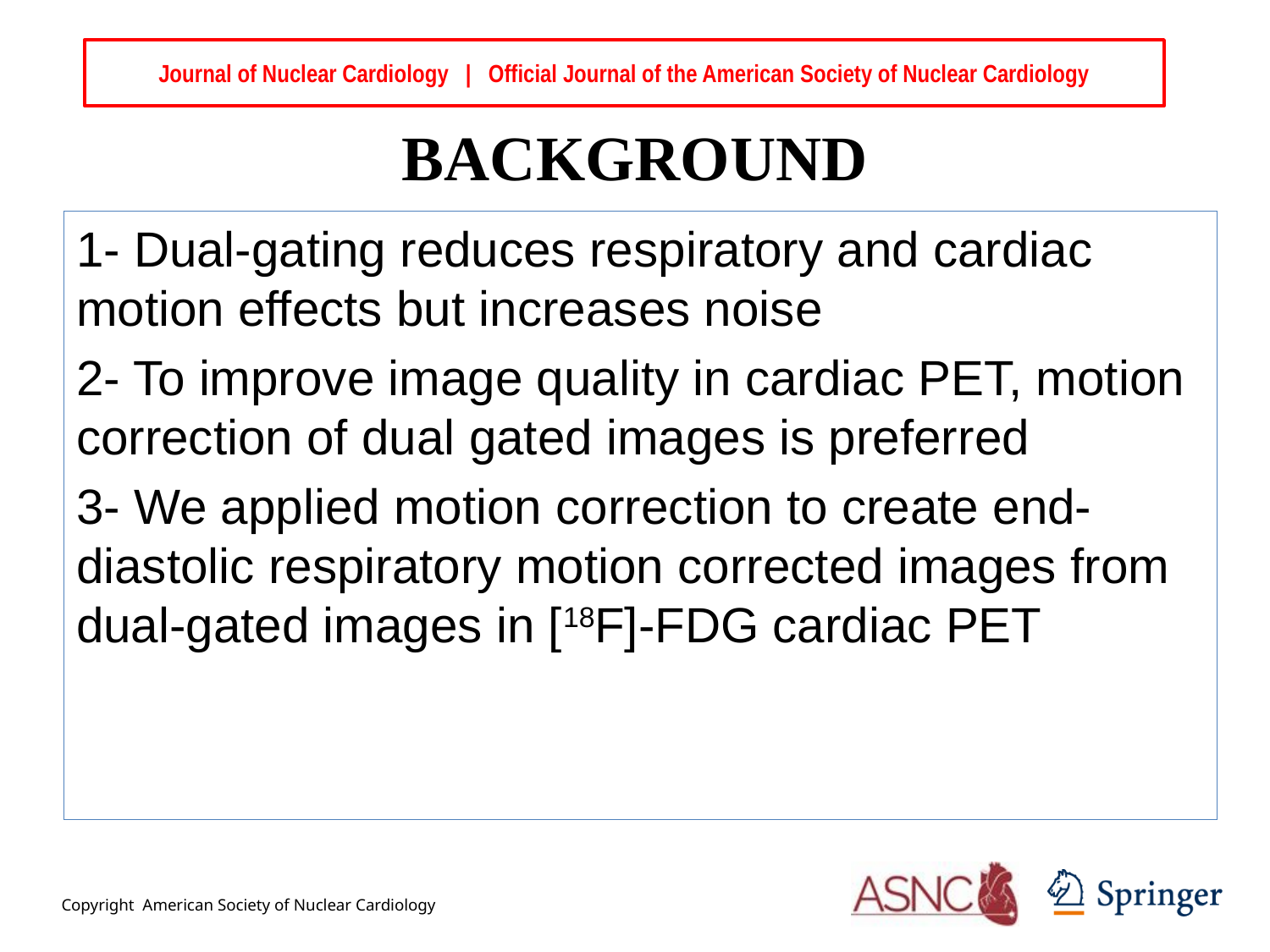

Journal of Nuclear Cardiology | Official Journal of the American Society of Nuclear Cardiology
# BACKGROUND
1- Dual-gating reduces respiratory and cardiac motion effects but increases noise
2- To improve image quality in cardiac PET, motion correction of dual gated images is preferred
3- We applied motion correction to create end-diastolic respiratory motion corrected images from dual-gated images in [18F]-FDG cardiac PET
Copyright American Society of Nuclear Cardiology

## Slide 3
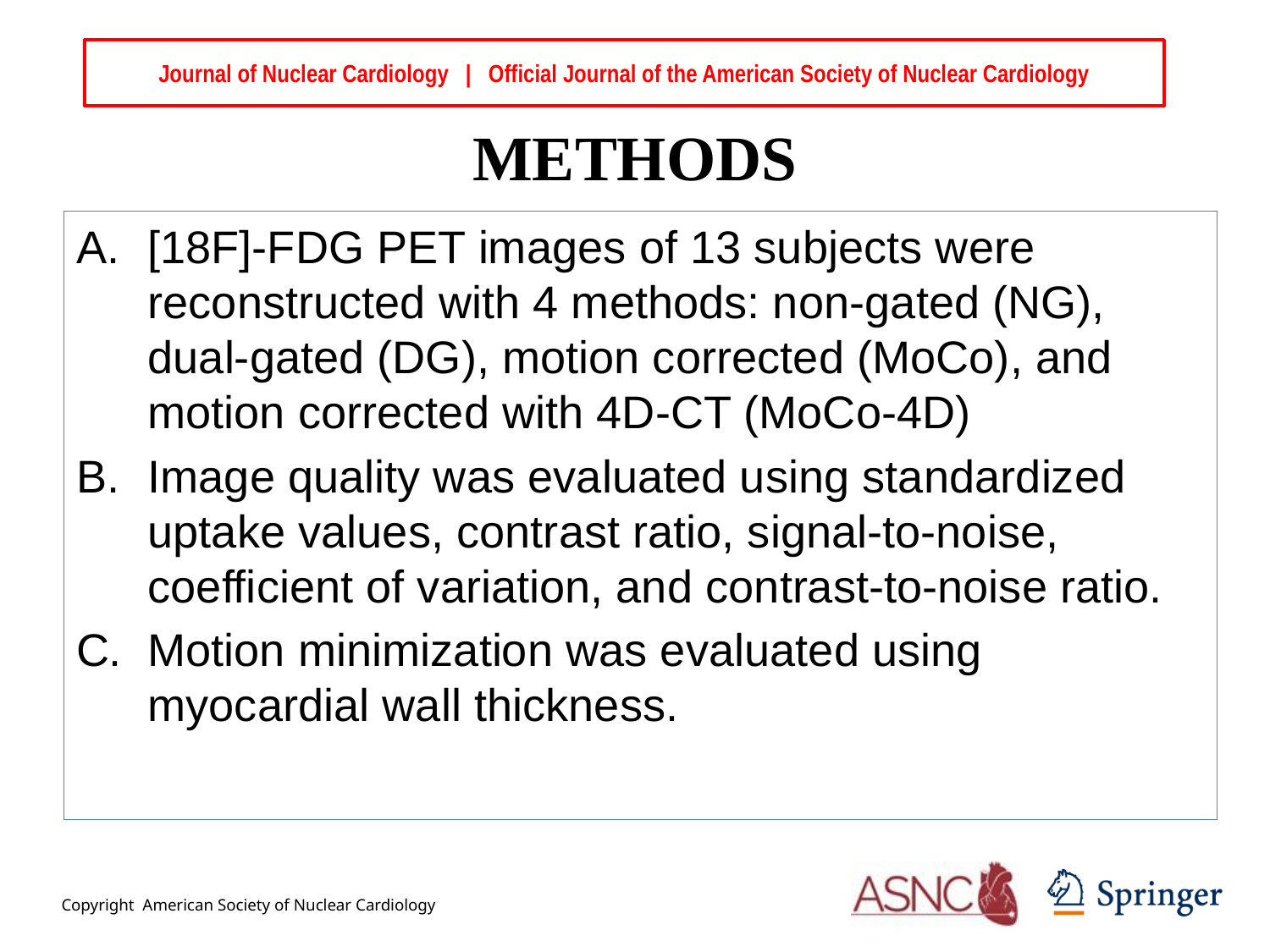

Journal of Nuclear Cardiology | Official Journal of the American Society of Nuclear Cardiology
# METHODS
[18F]-FDG PET images of 13 subjects were reconstructed with 4 methods: non-gated (NG), dual-gated (DG), motion corrected (MoCo), and motion corrected with 4D-CT (MoCo-4D)
Image quality was evaluated using standardized uptake values, contrast ratio, signal-to-noise, coefficient of variation, and contrast-to-noise ratio.
Motion minimization was evaluated using myocardial wall thickness.
Copyright American Society of Nuclear Cardiology

## Slide 4
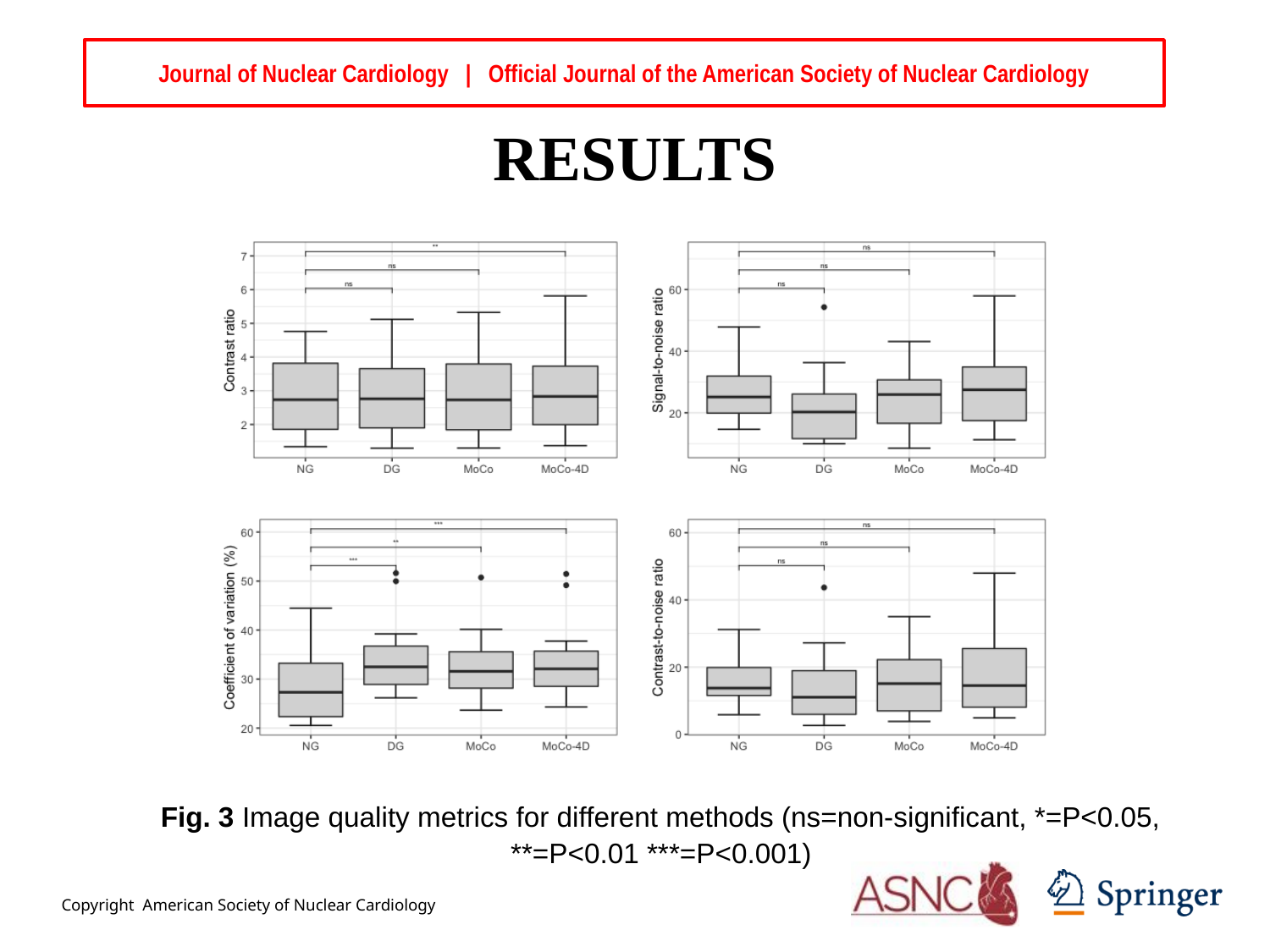

Journal of Nuclear Cardiology | Official Journal of the American Society of Nuclear Cardiology
# RESULTS
Fig. 3 Image quality metrics for different methods (ns=non-significant, *=P<0.05, **=P<0.01 ***=P<0.001)
Copyright American Society of Nuclear Cardiology

## Slide 5
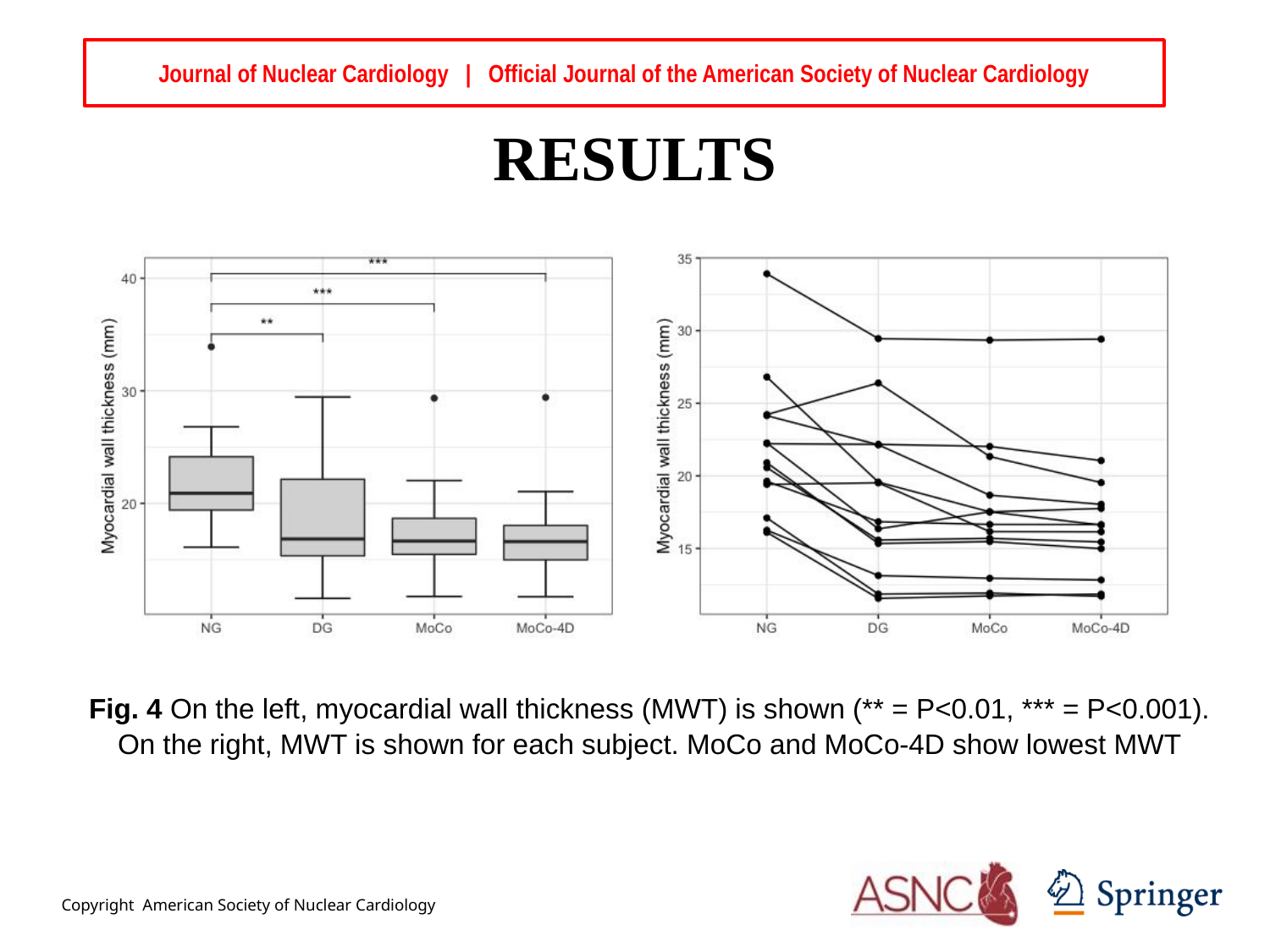

Journal of Nuclear Cardiology | Official Journal of the American Society of Nuclear Cardiology
# RESULTS
Fig. 4 On the left, myocardial wall thickness (MWT) is shown (** = P<0.01, *** = P<0.001). On the right, MWT is shown for each subject. MoCo and MoCo-4D show lowest MWT
Copyright American Society of Nuclear Cardiology

## Slide 6
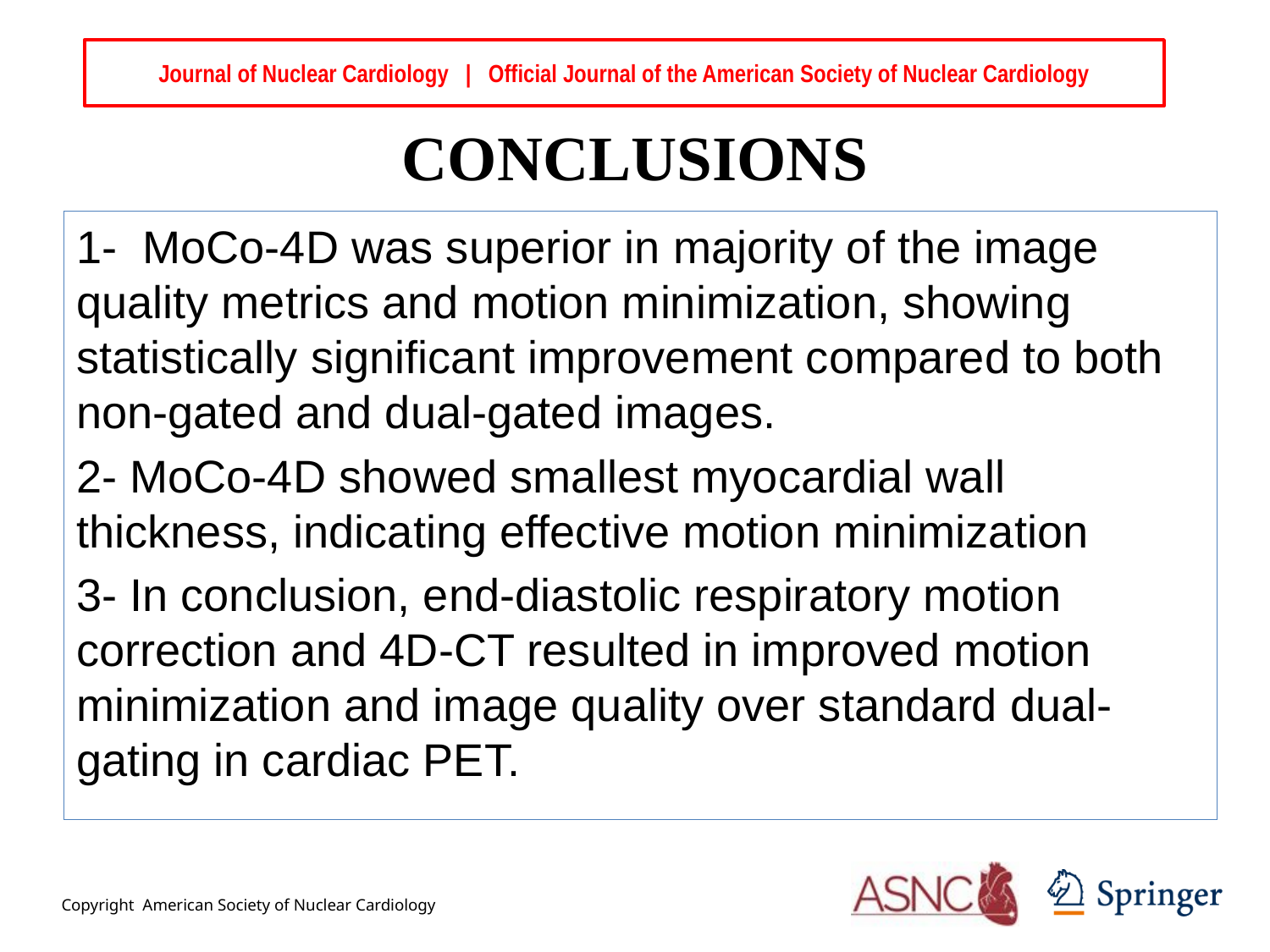

Journal of Nuclear Cardiology | Official Journal of the American Society of Nuclear Cardiology
# CONCLUSIONS
1- MoCo-4D was superior in majority of the image quality metrics and motion minimization, showing statistically significant improvement compared to both non-gated and dual-gated images.
2- MoCo-4D showed smallest myocardial wall thickness, indicating effective motion minimization
3- In conclusion, end-diastolic respiratory motion correction and 4D-CT resulted in improved motion minimization and image quality over standard dual-gating in cardiac PET.
Copyright American Society of Nuclear Cardiology
